# Charge injection based electrical stimulation on polypyrrole planar electrodes to regulate cellular osteogenic differentiation†

**DOI:** 10.1039/c8ra02601g

**Published:** 2018-05-21

**Authors:** Zongguang Liu, Lingqing Dong, Kui Cheng, Zhongkuan Luo, Wenjian Weng

**Affiliations:** School of Materials Science and Engineering, State Key Laboratory of Silicon Materials, Zhejiang University Hangzhou 310027 China wengwj@zju.edu.cn; The Affiliated Stomatologic Hospital, School of Medicine, Zhejiang University Hangzhou 310003 China; Zhejiang-California International NanoSystems Institute Hangzhou 310058 China

## Abstract

In this study, polypyrrole (Ppy) electrodes were prepared to support an electrical stimulation to MC3T3-E1 cells for regulating their osteogenic differentiation. The charge injection capacity (*C*_Q_) of the Ppy electrodes could be adjusted by the Ppy thickness, and a higher *C*_Q_ could make the electrode able to produce a higher charge injection quantity (*Q*_inj_) at applied voltage. The *Q*_inj_ onto electrode could be considered as the intensity of the stimulation pulse to cells, and the pulse frequency means the number of electric stimulation with *Q*_inj_ at one second. Hence, we conducted the present work in the view of *Q*_inj_. When the cells were electrically stimulated for 1 hour per day, the electrodes with *Q*_inj_ ranged in 0.08–0.15 μQ had an obvious role in enhancing cellular osteogenic differentiation whereas *Q*_inj_ of lower than 0.03 μQ or more than 0.30 μQ gave the stimulations with no or negative effects. And the stimulation with 1 or 25 Hz showed to enhance the differentiation, whereas the stimulation with 50 Hz gave an inhibiting effect. We further found the osteogenic differentiation potential triggered by electrical simulation was related to cell growth stage, and the stimulation carried out at early stage (day 2–5) during 8 days cell culture showed more contribution to enhancing osteogenic differentiation than that at later stage (day 6–8). It is proposed that the desired stimulation effects require that an appropriate voltage-gated calcium ion channel and efficient intracellular calcium ion oscillation are well activated. This work therefore reveals *Q*_inj_ as an important electrode parameter to decide effective simulations and provides an insight into understanding of the role of electrode material characters in regulating cellular osteogenic differentiation during stimulation.

## Abstract 2

This study reveals that the *Q*_inj_ on electrodes is a more significant factor than applied voltage for electrical stimulation to regulate cellular osteogenic differentiation, and the charge injection capacity can be tuned by thickness of Ppy.

## Graphical abstract

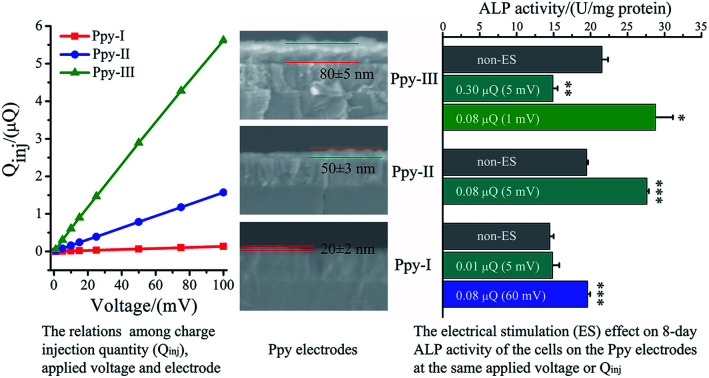

## 1 Introduction

Electric fields are believed to play a critical role in major biological processes such as embryogenesis, muscle contraction, wound healing and tissue regeneration.^1–3^ Inspired by the natural electrical properties of bone, an electric field stimulation generated by exogenous electric devices demonstrates a great potential to regulate osteogenic functions of stem and osteoblast-like cells and promote bone growth.^4–10^ To generate an effective electrical stimulation, the electric devices/electrodes and material selections are crucial. Compared with traditional parallel electrodes, planar interdigitated electrodes (IDE) integrate the pairing electrodes onto the same plane and generate stimulation under low voltage and in a direct, effective, highly reproducible and controlled manner.^11–13^

Various materials have been served as electrodes with different electric field intensities (or voltages) for electrical stimulation in osteogenic functions. For instance, McCullen *et al.* used a gold electrode with interdigitated shape to deliver stimulation under alternating electric field of 1 V cm^−1^, and the results showed an increase in cellular osteogenic differentiation potential of human adipose-derived stem cells (hASCs).^4^ An electrode material of conductive 3D silk foams produced stimulation with an interpenetrating network of poly (pyrrole-co-(2-hydroxy-5-sulfonic aniline)) bone tissue scaffolds with the electric field of 1 V cm^−1^, and had a potential to enhance the differentiation toward osteogenic outcomes of hMSCs.^14^ A Ti electrode covered by defined nanotubular TiO_2_ produced stimulation with constant electric field strength of 200 or 400 mV cm^−1^, which triggered the cellular osteogenic induction of mesenchymal stem cells (MSCs).^5^ An electrode material of conductive polypyrrole films supported stimulation with electric field of 350 mV cm^−1^, and showed its capability to promote osteogenesis of calcium deposition in the extracellular matrix of rat bone marrow mesenchymal stem cells (rBMSCs).^15^ Pelto *et al.* showed an enhanced early osteogenic differentiation of adipose stem cell proliferation on an electrode material of chondroitin sulfate (CS)-doped polypyrrole-coated polylactide scaffolds, which provided stimulation with DC voltage of 200 mV cm^−1^.^16^ Hardy *et al.* developed electrode materials of biomineralized conducting polymers and conductive polycaprolactone (PCL) fiber-based bone tissue scaffolds to enhance the differentiation towards osteogenic outcomes of hMSCs with the electric field of 100 mV cm^−1^.^17,18^ A moderately conductive artificial extracellular matrix coated polyaniline substrates was employed by Thrivikraman to apply stimulation with intermittently electric field of 3.6 mV cm^−1^, and the data showed an enhancement in osteogenic differentiation potential of human mesenchymal stem cells (hMSCs).^6^ It is evident from this vast literatures that a large range (1 V to 3.6 mV cm^−1^) of the applied voltage intensities were involved to stimulate to cells for osteogenic functions due to different electrode materials.

An electrode for electric simulation to cells is desired to elicit effective cellular responses at applied voltages as low as possible in order to avoid electrolysis and accompanying pH- and ionic-gradients in long-term experiments.^5,19,20^ For these electrodes, electrical properties would be of greater concern.^21–23^ The interaction ability of the electrodes with external species is believed to be proportional to electrical property of charge injection capacity (*C*_Q_) of electrodes at a given applied voltage.^24^ Therefore, it is pursued for a material to support an electrode with high *C*_Q_.

In this study, conductive polymer polypyrrole (Ppy) was selected as electrode material due to its reversible-faradaic reactivity which resulted in a high *C*_Q_. Ppy planar interdigitated electrodes were prepared by electro-polymerization, and the *C*_Q_ of electrodes was adjusted by changing Ppy polymerized conditions. An electrical stimulation with biphasic pulse mode was performed during culturing of MC3T3-E1. In the view of charge injection, the effect of electrical stimulation on the cellular osteogenic differentiation was studied at different applied voltages and stimulation times (the pulse frequency and the total stimulation time). Moreover, the possible mechanism of electrical stimulation to cells was also discussed.

## 2 Materials and methods

### 2.1. Preparation of Ppy electrodes

**Fig. 1:**
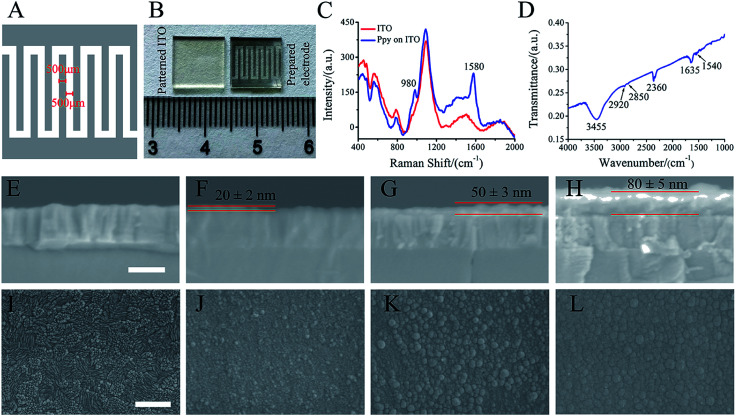
Characterization of Ppy electrodes prepared by electrochemical polymerization. The interdigitated electrode (10 mm × 10 mm) with 500 μm width and spacing (A). The photograph of patterned ITO substrate (left) and Ppy electrode (right) prepared by electrochemical polymerization (B). The Raman spectrum (C) and FTIR (D) of the Ppy electrodes; thickness of the patterned ITO substrate (E) and Ppy electrodes prepared at current density of 3 mA cm^−2^ (F), 5 mA cm^−2^ (G), and 8 mA cm^−2^ (H); surface morphologies of the patterned ITO substrate (I) and Ppy electrodes prepared at current density of 3 mA cm^−2^ (J), 5 mA cm^−2^ (K), and 8 mA cm^−2^ (L). (E–H) shares the same scale bar of 200 nm, (I–L) shares the same scale bar of 500 nm.

ITO glass (10 mm × 10 mm) was patterned by laser etching to form 500 μm width ITO strip and spacing (Fig. 1A). The electro-polymerization of Ppy was conducted with a two-electrode electrochemical system under galvanostatic conditions. The patterned ITO substrate and platinum sheet were used as a working and counter electrodes, respectively. The current densities of 3 mA cm^−2^, 5 mA cm^−2^ and 8 mA cm^−2^ with 30 s were utilized to electro-polymerize for forming Ppy electrodes with different Ppy thickness. As-formed electrodes were washed with deionized water and followed by air-drying overnight. The electro-polymerization solution was consisted of 0.1 M pyrrole (Sigma-Aldrich, USA) and 0.1 M sodium *p*-toluene sulfonic acid (*p*TS) (Macklin, China).

### 2.2. Characterizations of Ppy electrodes

Raman spectra of the prepared electrodes on patterned ITO substrate were taken by OMNIC Dispersive Raman (Thermo Fisher Scientific, USA) with a DXR laser operating at 532 nm with incident power of 10 mW. The prepared electrode materials were peeled from patterned ITO substrate and mixed with KBr, and then FTIR spectrum was collected in the range of 4000–1000 cm^−1^. The thickness and morphologies of the prepared electrodes were examined by Field Emission Scanning Electron Microscopy (FESEM; Hitachi, SU-70). Prior to SEM analysis, all prepared electrodes were sputter-coated with a thin layer of gold.

### 2.3. Electrochemical analysis

All electrochemical experiments were conducted by two-electrode system in culture medium using a computer-controlled CHI 660D electrochemical workstation (Chenhua Instrument Co., Shanghai, China). The culture medium was consisted of alpha-modified Minimum Essential Medium (Alpha-MEM, Gibco, Waltham, MA) and 10% fetal bovine serum (Gibco) and 1% antibiotics (10 000 units per mL penicillin and 10 000 μg mL^−1^ streptomycin, Gibco). As a planar interdigitated electrode, one side of Ppy electrode was used as working electrode and another as counter electrode. Cyclic voltammetric (CV) measurements were performed from 1 V to −1 V at a scan rate of 50 mV s^−1^. The current–time curves were collected under a biphasic pulses signal (used in electrical stimulation experiments) with different voltages. Then the current was integrated over time in the charged period in each curve using Originpro 8.5 software. The obtained integral area was the *Q*_inj_ (the total amount of charge injected on the electrode during a stimulus pulse) on electrode (Fig. S2†).^25^ Finally, *Q*_inj_–voltage curves were drawn according to the calculated values.

### 2.4. Cell spreading, viability and proliferation

Ppy electrode was placed in a home-made culturing device (made by polytetrafluoroethylene, shown in Fig. S1†), and then Mouse calvaria-derived pre-osteoblastic cells (MC3T3-E1) were seeded at a density of 2 × 10^4^ cells per cm^2^ and maintained at 37 °C and 5% CO_2_ in a humidified atmosphere.

Cellular attachment and spreading on Ppy electrodes after 1 day of culture was determined by using immunofluorescence staining. In addition, typical morphology of cells on Ppy electrodes after 2 days of stimulation was also observed. The cells on the electrodes were fixed with paraformaldehyde (4% in PBS) for 15 min, and then permeabilized with 0.4% Triton X 100 for 15 min, blocked in solution contained BSA and FBS (2% in PBS) for 1 hour. After washing in PBS, cells were incubated with fluorescent dye of anti-vinculin (EPR8185, Abcam, UK), rhodamine phalloidin (Phalloidin-iFluor™ 594 Conjugate, AAT Bioquest, Inc. USA) and 4′,6-diamidino-2-phenylindole (DAPI, ENZ-52404, Enzo Life Sciences, Switzerland). The vinculin (green), cytoskeleton (red) and nucleus (blue) were visualized by confocal laser scanning microscopy (Zeiss LSM 780, Germany).

The viability and proliferation of the cells cultured on Ppy electrodes were determined by the Cell Counting Kit-8 (CCK-8, Dojindo Laboratories, Kumamoto, Japan). Briefly, cells on Ppy electrodes were transferred to a new 24-well plate after culture for 1 and 5 days, and then 500 μL fresh culture media and 50 μL of CCK-8 solution were added to each well. After incubated for 3 h at 37 °C, the solution was dispensed into a 96-well plate, and colorimetric measurements of formazan dye were conducted with the microplate reader at 450 nm.

### 2.5. Electrical stimulation

Before seeding cells, Ppy electrode was placed into a home-made culturing device (Fig. S1†), and then two platinum wires were fixed onto the two conductive sides in an electrode by polytetrafluoroethylene screws. MC3T3-E1 cells were seeded at a density of 2 × 10^4^ cells per cm^2^, and all stimulations to cells were performed after 1 day pre-culture to permit cellular attachment and spreading. The two wires were connected to a waveform generator (DG1022 type, Rigol Electronic Co., Ltd., Beijing, China) and the stimulation was applied on cells under a biphasic pulse with 10 ms pulse duration.

The Ppy electrodes were conducted to stimulate cells under the applied voltages of 5 mV or 25 mV at 1 Hz, trying to understand the role of *Q*_inj_. The electrodes with the same *Q*_inj_ were conducted to stimulate cells under different voltages calculated by charge–voltage curves. The stimulation frequencies of 1 Hz, 25 Hz or 50 Hz with the applied voltages of 5 mV were adopted to stimulate cells on the Ppy electrodes. The cell culture period of 8 days was divided into two stimulation stages, the early stage (day 2–5) and later stage (day 6–8). The stimulation to cells on the electrodes at the two stages was performed with applied voltage of 5 mV. The cells on the Ppy electrodes without electrical stimulation (ES) were used as control (non-ES).

### 2.6. Alkaline phosphatase analysis

After 8 days of culture, culture medium was removed, and the cells on Ppy electrodes were rinsed with PBS for three times. The cells were lysed with CelLytic Buffer (Sigma, St. Louis), and the obtained cell lysate was centrifuged with the speed of 12 000 rmp at 4 °C for 15 min. The supernatants were assayed by LabAssay™ ALP (Wako Pure Chemical Industries, Ltd. Japan) *via* measuring the optical density at a wavelength of 405 nm. The ALP activities were obtained by normalizing the quantitative assay values to total protein contents tested in a BCA protein assay.

### 2.7. Quantitative real-time PCR assay

The expression of osteogenesis-related genes (ALP, Runx2 and OCN) and Ca^2+^-calcineurin/NFAT signal genes (calmodulin (CaM), calcineurin (CaN), and NFAT) was analyzed by real-time (RT) polymerase chain reaction (PCR) assay. After 8 days of culture, the total RNA was extracted using TRIzol reagent and further collected using the miRNeasy Mini Kit (QIAGEN 217004, USA). RNA samples were reverse transcribed to cDNA in reactions using the PrimeScript™ RT reagent Kit with gDNA Eraser (Perfect Real Time) (Takara RR047A, Japan) according to manufacturer's protocol. The qPCR reactions were conducted on the Mastercycler® ep realplex system (Eppendorf, Germany) with a SYBR Green (PowerUp™ SYBR™ Green Master Mix (Applied Biosystems A25742, USA)) using 40 cycles at 95 °C for 2 min, 60 °C for 30 s, then 72 °C for 30 s and were performed in triplicate for each cDNA. The relative expression of genes was normalized to that of the reference gene β-actin. The following primer pairs related to the targeted RNA were used in this study: β-actin (5′-AATGTGGCTGAGGACTTTG-3′ and 5′-GGGACTTCCTGTAACCACTTATT-3′); ALP (5′-CCAGAAAGACACCTTGACTGTGG-3′ and 5′-TCTTGTCCGTGTCGCTCACCAT-3′); OCN (5′-GCAATAAGGTAGTGAACAGACTCC-3′ and 5′-CCATAGATGCGTTTGTAGGCGG-3′); Runx2 (5′-CCTGAACTCTGCACCAAGTCCT-3′ and 5′-TCATCTGGCTCAGATAGGAGGG-3′); CaM (5′-GG GTCAGAACCCAACAGAAG-3′ and 5′-GTCAAGAACTCTGGGAAGTCAA-3′); CaN (5′-GTAGGCACCTCACAGAGTATTT-3′ and 5′-CAGTCGAAGGCATCCATACA-3′); NF-AT (5′-CCGTCCAAGTCAGTTTCTATGT-3′ and 5′-GTCCGTGGGTTCTGTCTTTAT-3′).

### 2.8. Measurement of intracellular Ca^2+^

Intracellular Ca^2+^ levels were measured using Fluo4-AM calcium indicator (Dojindo Laboratories, Japan) after 2 days of stimulation or stimulation at later stage (day 4–5). After stimulation, the cells on Ppy electrodes were rinsed with HBSS and the 400 μL of Fluo4-AM dye loading solution (4 μM in HBSS) was added. The plates were incubated at 37 °C for 30 minutes for the complete reaction. Successively, the cells were washed with HBSS and collected by 0.5 mL trypsin. After three times of centrifugation, mean fluorescence intensity of cell suspension was measured by flow cytometer (Cytoflex, Beckman Coulter, China) with the emission wavelength at 518 nm and exaction wavelength at 495 nm.

### 2.9. Statistical analysis

All the quantitative data in this study were expressed as mean ± standard deviation (S.D.). Statistical analysis was performed using the software of Statistical Package for the Social Sciences (SPSS) version 19. The significant differences were performed by one-way analysis of variance (ANOVA) followed by Tukey's test. In all of the statistical evaluations, the differences with *p* < 0.05 were considered as statistically significant.

## 3 Results

### 3.1. Characterizations of Ppy electrodes

As shown in Fig. 1A, the patterned electrode was interdigitated shape (10 mm × 10 mm) with 500 μm width and spacing. Fig. 1B shows the photograph of patterned ITO substrate (left) and the prepared electrode (right) that obtained by electrochemical polymerization. Obviously, black materials with interdigitated shape were found after polymerization.

As shown in Fig. 1C, the obtained electrode exhibits typical Raman peaks at 1580 and 980 cm^−1^, which are assigned to the symmetric C=C backbone stretching mode and C–H plane deformation.^26–29^ The FTIR spectrum (Fig. 1D) shows the broad band around 3000–3500 cm^−1^, which could be ascribed to N–H and aromatic C–H stretching vibrations. The two absorption peaks at 2851 cm^−1^ and 2922 cm^−1^ are corresponded to symmetric and asymmetric stretching vibrations of C–H groups, respectively. The absorption peaks at 2360 cm^−1^ is related to stretching vibrations of C–N. The bands at 1635 cm^−1^ and 1540 cm^−1^ are corresponded to C=C ring stretching vibration of pyrrole ring and benzene ring of dopant (pTS).^30–32^ The Raman and FTIR spectra of the prepared electrode indicated that Ppy was formed on the patterned ITO substrate.

The SEM images of the electrode cross-section (Fig. 1F–H) show that the thickness of the Ppy electrodes on patterned ITO substrate (Fig. 1E) by electro-polymerization increased with current densities. The electrode thickness of 20 ± 2 nm, 50 ± 3 nm and 80 ± 5 nm corresponded to the electro-polymerization at current densities of 3 mA cm^−2^, 5 mA cm^−2^ and 8 mA cm^−2^, respectively. Fig. 1J–L shows the morphologies of the obtained electrodes at different current densities. Ppy grains were observed to homogeneously cover the patterned ITO substrate (Fig. 1I) at current density of 3 mA cm^−2^ (Fig. 1J), and the grains became large at current density of 5 mA cm^−2^ (Fig. 1K). Further increasing the current density to 8 mA cm^−2^, the large grains demonstrated a smoother and denser surface morphology (Fig. 1L). The Ppy electrodes prepared at current density of 3 mA cm^−2^, 5 mA cm^−2^ and 8 mA cm^−2^ were named by Ppy-I, Ppy-II and Ppy-III, respectively.

### 3.2. The *C*_Q_ and *Q*_inj_ of Ppy electrodes

**Fig. 2:**
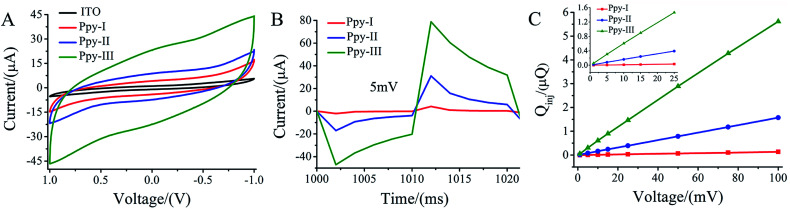
Electrochemical analysis of Ppy electrodes in culture medium. (A) Cyclic voltammetric (CV) measurement; (B) current–time curve collected with a pulse signal; (C) the *Q*_inj_–voltage curve of Ppy electrodes calculated from the time integral of the current in (B).

The CV curves (Fig. 2A) demonstrate that three typical electrodes exhibit different loops within the voltage window ranging from 1 to −1 V in culture medium. The enclosed area in CV curve with the overall voltage range represents charge injection capacity (*C*_Q_) of the electrodes,^33^ and *C*_Q_ increased with polymerized current densities. Obviously, the patterned ITO substrate demonstrated a much lower *C*_Q_ compared to that of Ppy electrodes.

Fig. 2B shows the electric current responses of Ppy-I, Ppy-II and Ppy-III electrodes to the electric voltage pulses at 5 mV. The curve area of time integral of the current in charging period was considered to be the quantity of electric charge injected onto the electrodes, *i.e. Q*_inj_ (Fig. S2†).^25^Fig. 2C shows the *Q*_inj_ obviously increased upon the increasing of electrode *C*_Q_ and applied voltage.

### 3.3. Cell adhesion, spreading, viability and proliferation on Ppy electrodes

**Fig. 3:**
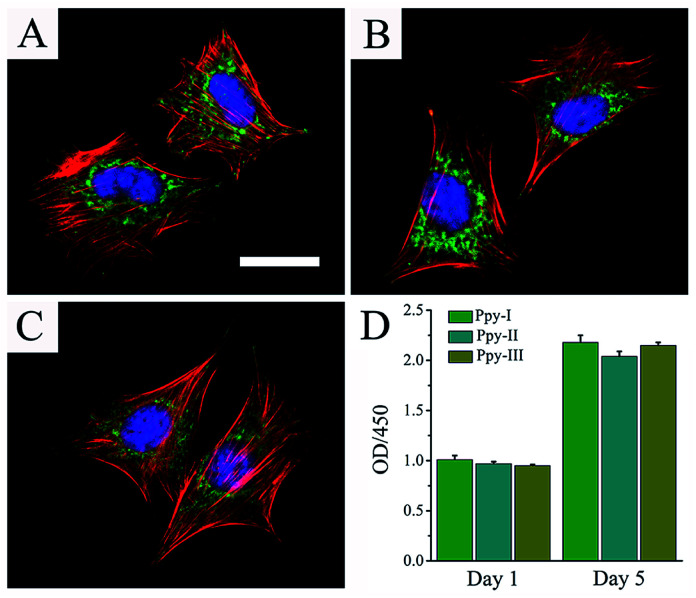
Cellular morphologies on the Ppy electrodes were observed by immunofluorescent staining. (A) On Ppy-I; (B) on Ppy-II; (C) on Ppy-III. Cells were stained for the FA protein vinculin (green), cellular nuclei (blue) and actin cytoskeleton (red). (A–C) shares the same scale bar of 100 μm. Cellular viability and proliferation on the Ppy electrodes were evaluated by CCK-8 (D).

Fig. 3A–C shows the cellular morphologies on the prepared electrodes indicated by immunofluorescent staining after 1 day of culture. F-actin and vinculin indicated the cytoskeleton-related proteins associated actin and integrins, respectively. The organization of actin and expression of vinculin throughout the spreading regions of cells grown on all Ppy electrodes confirmed the cytocompatibility of the electrodes. The results revealed that the prepared Ppy electrodes were suitable for cell adhesion and spreading.

Fig. 3D shows that cells on different electrodes presented the similar viability after 1 day of culture and cellular proliferation increased significantly after 5 days of culture. These results indicated the excellent adhesion and biocompatibility of the electrodes.

### 3.4. The ES effects of stimulation intensity on osteogenic differentiation

For electrical stimulation to cells, 5 mV and 25 mV for applied voltages were selected because the voltages could give the Ppy electrodes to have significant difference of *Q*_inj_ in three orders of magnitude according to the *Q*_inj_–voltage curve (Fig. 2C).

**Fig. 4:**
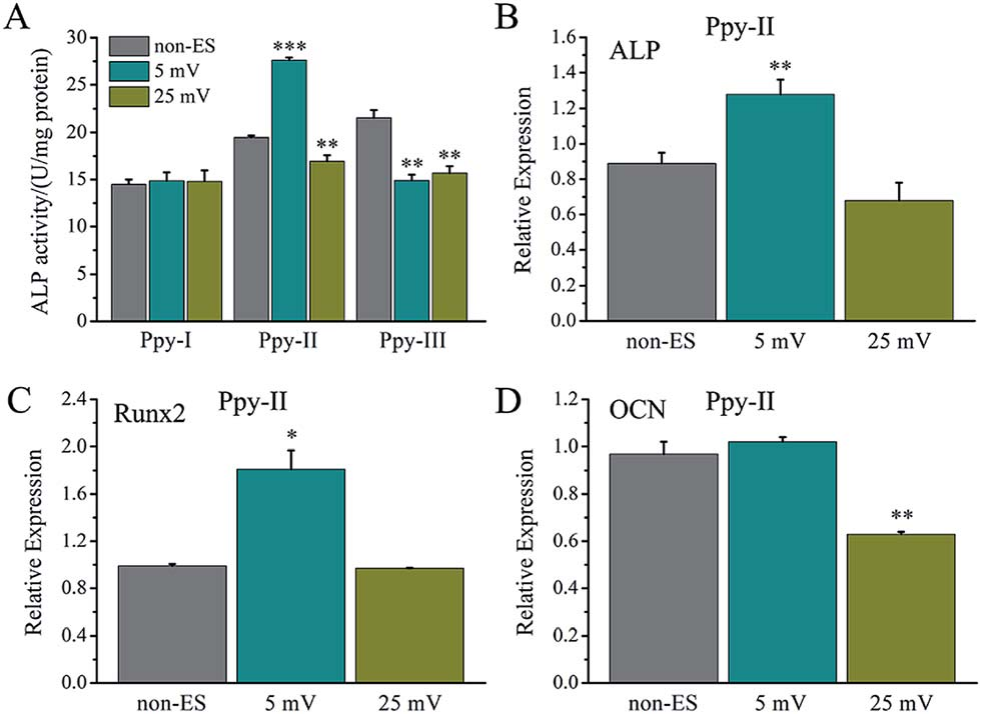
The ES effect of the applied voltages on osteogenic differentiation of the cells on Ppy electrodes. (A) ALP activity on Ppy electrodes with different voltages; (B–D) osteogenesis-related gene expressions on Ppy-II with applied voltage of 5 mV (0.08 μQ) and 25 mV (0.30 μQ). *p < 0.05, **p < 0.01, ***p < 0.001.

As shown in Fig. 4A, at the applied voltage of 5 mV, Ppy-I electrode with *Q*_inj_ of 0.01 μQ showed no effect on osteogenic differentiation due to low *C*_Q_. Ppy-II electrode with *Q*_inj_ of 0.08 μQ expressed a promoted differentiation, while Ppy-III electrode with *Q*_inj_ of 0.30 μQ inhibited the differentiation due to high *C*_Q_. At the applied voltage of 25 mV, Ppy-I electrode with *Q*_inj_ of 0.03 μQ still showed no effect on osteogenic differentiation, whereas Ppy-II electrode with *Q*_inj_ of 0.40 μQ and Ppy-III electrode with *Q*_inj_ of 1.5 μQ inhibited differentiation. Those indicate the Ppy electrode with high *C*_Q_ at low applied voltage is able to enhance differentiation.

Fig. 4B–D shows the osteogenesis-related gene expressions of the cells stimulated on Ppy-II electrode with *Q*_inj_ of 0.08 μQ (5 mV) and 0.30 μQ (25 mV). The results showed that the gene expressions of ALP and Runx2 were up-regulated for *Q*_inj_ of 0.08 μQ, whereas the simulation with *Q*_inj_ of 0.30 μQ showed no effect on ALP and Runx2 expression, and down-regulated the expression of OCN significantly.

**Fig. 5:**
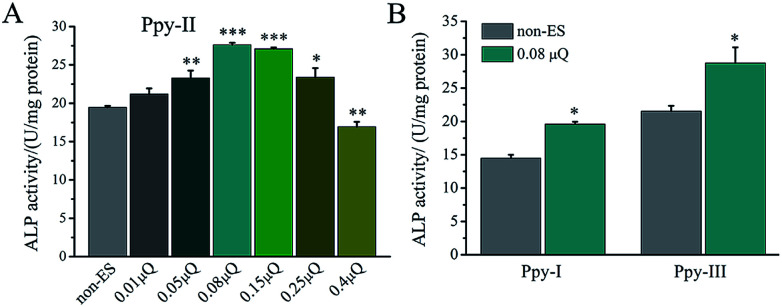
The ES effect of the *Q*_inj_ on osteogenic differentiation of the cells on Ppy electrodes. (A) ALP activity on Ppy-II electrodes with different *Q*_inj_; (B) ALP activity on Ppy-I and Ppy-III electrodes with *Q*_inj_ of 0.08 μQ. *p < 0.05, **p < 0.01, ***p < 0.001.

The effect of *Q*_inj_ on differentiation on Ppy-II electrode was shown in Fig. 5A. ALP activity increased gradually with *Q*_inj_ increasing, and *Q*_inj_ of 0.08 μQ showed the largest ALP activity. The *Q*_inj_ of 0.4 μQ decreased the ALP activity significantly.

When *Q*_inj_ of 0.08 μQ was set for other two Ppy electrodes using different applied voltages, Ppy-I (60 mV) and Ppy-III (1 mV) electrodes presented the same stimulation effect with Ppy-II electrode (5 mV) for enhancing osteogenic differentiation (Fig. 5B). Obviously, Ppy-II electrode with *Q*_inj_ of 0.08 μQ (5 mV) showed the most significant upregulation of osteogenic differentiation compared with Ppy-I and Ppy-III electrodes (Fig. 5A), which was selected for further studies.

### 3.5. The ES effects on cell cytoskeleton

When cells were seeded onto the Ppy electrode, the cells grew to homogenously distribute on whole electrode including conducting Ppy and spacing glass strips after 2 days of stimulation, this could result from that all cells on the electrode received the equal stimulation effect.

**Fig. 6:**
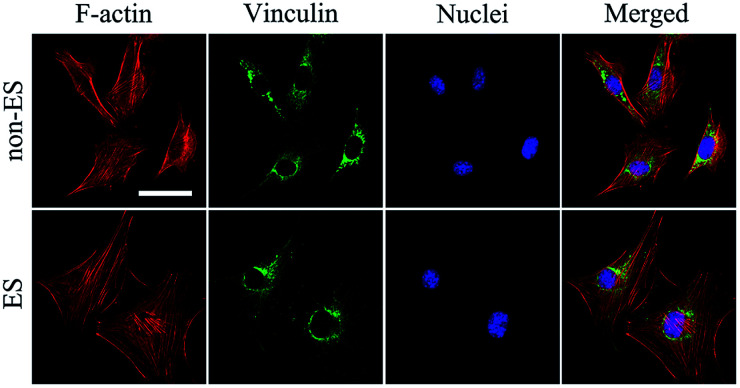
Cellular morphologies on the Ppy-II were indicated by immunofluorescent staining after 2 days of stimulation (0.08 μQ, 5 mV). Cells were stained for the FA protein vinculin (green), cellular nuclei (blue) and actin cytoskeleton (red). The images share the same scale bar of 50 μm.

Fig. 6 shows the cellular cytoskeleton on the Ppy-II indicated by immunofluorescent staining after 2 days of stimulation under *Q*_inj_ of 0.08 μQ (5 mV). The spreading area of actin and expression of vinculin in stimulation group was larger than that of non-stimulated group. The results indicated that electrical stimulation promoted the cellular spreading and migration on Ppy electrode.

### 3.6. The ES effect of stimulation time on cellular osteogenic differentiation

For electrical stimulation to cells at a biphasic electric pulse mode, the frequency of electric pulses means a stimulating pulse number in one second, and total stimulation time represents a stimulating day number in the whole cell culture period.

**Fig. 7:**
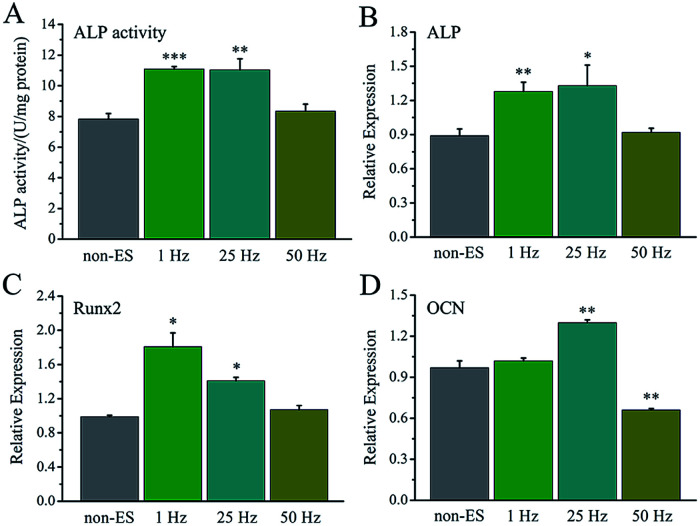
The ES effect of the stimulating pulse frequency on osteogenic differentiation of the cells on Ppy-II electrodes. (A) ALP activity; (B) ALP gene expression; (C) Runx2 gene expression; (D) OCN gene expression. *p < 0.05, **p < 0.01, ***p < 0.001.

The effect of stimulating frequency on osteogenic differentiation for 1 hour stimulation per day was showed in Fig. 7A, 1 Hz or 25 Hz demonstrated to enhance the differentiation, whereas 50 Hz inhibited the differentiation. Fig. 7B–D shows the osteogenesis-related gene expressions under the stimulation with different frequency, the cellular gene expressions of ALP, Runx2 and OCN stimulated with 1 Hz or 25 Hz were up-regulated, especially OCN expression. However, the stimulation with 50 Hz showed no effect on ALP and Runx2 expressions, but down-regulated OCN expression.

**Fig. 8:**
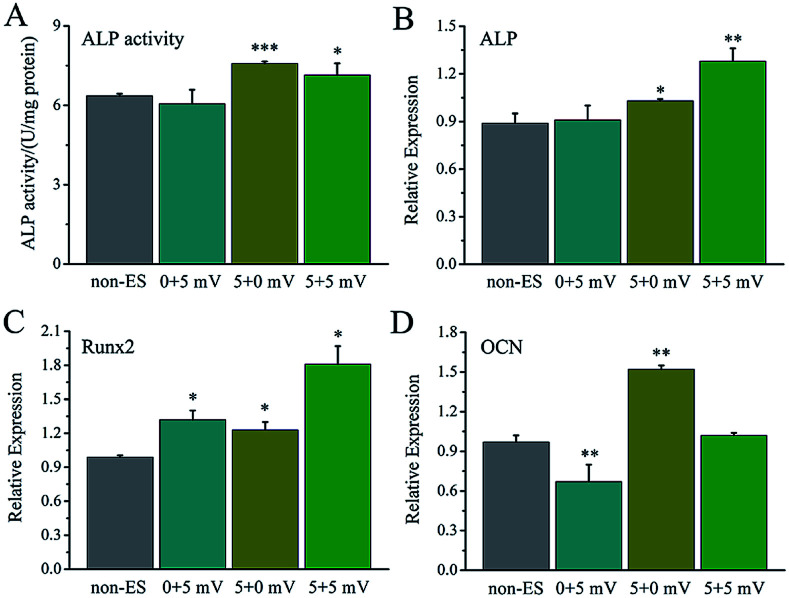
The ES effect of the stimulating days on osteogenic differentiation of the cells on Ppy-II electrode during 8 days cell culture (5 mV and 1 Hz), early stage (day 2–5) + later stage (day 6–8). (A) ALP activity; (B) ALP gene expression; (C) Runx2 gene expression; (D) OCN gene expression. *p < 0.05, **p < 0.01, ***p < 0.001.

The electrical stimulation carried out at early stage (day 2–5) showed more significant contribution to promotion of cellular osteogenic differentiation than that at early stage (day 6–8) in 8 day culture period (Fig. 8A), and a similar ALP level to that for 8 days.

The osteogenesis-related gene expressions of ALP (Fig. 8B), Runx2 (Fig. 8C) and OCN (Fig. 8D) that stimulated at early stage (day 2–5) were up-regulated. The stimulation carried out at later stage (day 6–8) showed no up-regulation of ALP gene expression, up-regulation of Runx2 and down-regulation of OCN.

### 3.7. The ES effect on Ca^2+^-calcineurin/NFAT signal pathway

**Fig. 9:**
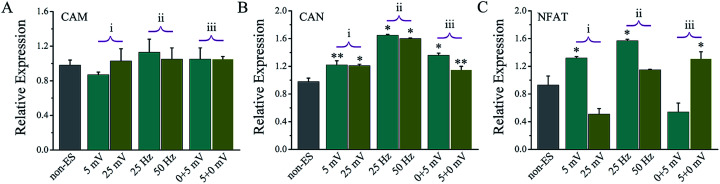
The ES effects on the gene expressions of CaN/NFAT signal pathway of the cells on Ppy-II electrode during 8 days culture. (i) The effect of the applied voltage (1 Hz and the stimulation at day 2–8); (ii) the effect of the frequency (5 mV and the stimulation at day 2–8); (iii) the effect of the stimulating days (early stage at day 2–5 + later stage at day 6–8, 1 Hz). *p < 0.05, **p < 0.01.

Fig. 9 shows the gene expressions of CaN/NFAT signal pathway of the cells stimulated on Ppy-II electrode with *Q*_inj_ of 0.08 μQ (5 mV) or 0.30 μQ (25 mV) and 25 Hz or 50 Hz and stimulation at early or later stage.

For the stimulated cells, their CaM gene expression showed no significant change, and CaN gene expression was up-regulated. As the last downstream gene of Ca^2+^ signal pathway, NFAT expression of the cells that stimulated with *Q*_inj_ of 0.08 μQ and 25 Hz or at early stages showed a significant up-regulation.

### 3.8. The ES effect on intracellular Ca^2+^

**Fig. 10:**
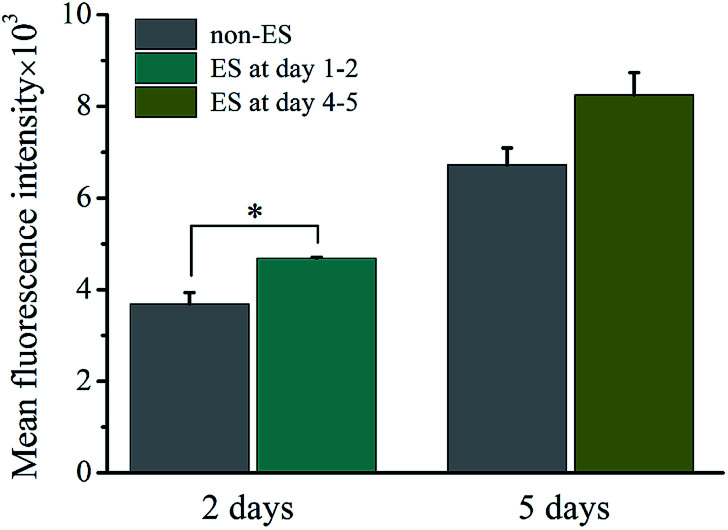
Intracellular Ca^2+^ concentration indicated by the mean fluorescence intensity which measured by flow cytometry. The stimulation conducted to cell on Ppy-II with *Q*_inj_ of 0.08 μQ at the applied voltage of 5 mV after stimulation of 2 days or stimulation at later stage (day 4–5).

Fig. 10 shows the intracellular Ca^2+^ concentration indicated by the mean fluorescence intensity (MFI) after 2 of stimulation or stimulation at later stage (day 4–5) on cell on Ppy-II with *Q*_inj_ of 0.08 μQ at the applied voltage of 5 mV.

The flow cytometry assay showed that intracellular Ca^2+^ concentration increased with culture time from 2 days to 5 days without stimulation. And after 2 days of stimulation, intracellular Ca^2+^ concentration increased significantly, whereas stimulation at later stage did not affect the Ca^2+^ concentration.

## 4 Discussion

The Ppy electrodes showed that their thickness increased with electro-polymerization current densities (Fig. 1). As a result, higher thickness led the electrode have higher *C*_Q_ (Fig. 1 and 2A) due to more spaces to accommodate electric charges derived from irreversible faradaic reaction.^34^

For a given applied voltage, the electrode with higher *C*_Q_ had a higher response to an applied voltage pulse (Fig. 2B) as well as a higher charge injection quantity (*Q*_inj_) (Fig. 2C) than those with lower *C*_Q_. *Q*_inj_ of the electrodes could be also tuned by the applied voltage. The electrodes with higher *Q*_inj_ means to induce a stronger electrical simulation pulse intensity to cells (Fig. 2C), and could give a stronger stimulation effect at lower applied voltage.

During electrical stimulations to cells, electric fields, currents and electrochemical factors derived from electrodes will contribute to the stimulation. In this study, we focused on the effects of electric field because there were no electrical current flow in Ppy stripes of the electrode under the present applied voltage and no redox peaks observed (Fig. 2A).

The Ppy film was testified to be very stable on the patterned ITO substrate for soaking in culture medium and showed to well support cellular adhesion, spreading and proliferation, good cytocompatibility (Fig. 3). The assay of cellular osteogenic differentiation (Fig. 4A) showed that the Ppy electrodes with higher *C*_Q_ produced significantly upregulated effect at lower applied voltage as expected.

Since the stimulation pulse intensity is directly related to *Q*_inj_, the stimulation effects of the electrodes could be evaluated by their *Q*_inj_. The result (Fig. 4A) showed that no or negative simulation effect on cellular osteogenic differentiation was generated for the Ppy electrode with *Q*_inj_ of low than 0.03 μQ (Ppy-I at 5 mV) or more than 0.30 μQ (Ppy-III at 5 mV), while the Ppy electrode with *Q*_inj_ of 0.08 μQ (Ppy-II at 5 mV) produced the simulation to have an obvious role in enhancing osteogenic differentiation as well as the up-regulated the expression of osteogenesis-related genes (Fig. 4B–D). When the Ppy-I or Ppy-III electrode was applied by 60 mV or 1 mV voltage to have *Q*_inj_ of 0.08 μQ, the two electrodes also demonstrated the obvious stimulation effect to enhance the differentiation (Fig. 5B). Hence, the Ppy electrode with higher *C*_Q_ and the stimulation pulse intensity of *Q*_inj_ of 0.08 μQ were appropriate to generate effective stimulations to cells for attaining the enhancement of osteogenic differentiation. In addition, this electrical stimulation promoted the cellular spreading and migration on electrode (Fig. 6).

For the electrical stimulation to cells, the stimulation time is another important parameter besides stimulation intensity of *Q*_inj_. The time could be divided into three parts, the time at one second with pulse number (the stimulation pulse frequency), the time at one day with stimulating hour (the daily stimulation time), and the time at the whole cell culture period with stimulating days (total stimulation time). The role of the daily stimulation time had been discussed in our previous work,^11^ and 1 hour per day showed appropriate in promoting cellular osteogenic differentiation, and was adopted in this study.

When *Q*_inj_ of Ppy-II electrode was set at 0.08 μQ (5 mV), the stimulation pulse frequency and total stimulation time were changed, the simulation pulse with 1 Hz or 25 Hz showed to enhance the differentiation, whereas that with 50 Hz inhibited the differentiation and osteogenesis-related gene expressions (Fig. 7). During cell culture for 8 days, the electrical stimulation carried out at early stage (day 2–5) demonstrated more obvious role in promoting cellular osteogenic differentiation than that at later stage (day 6–8) (Fig. 8). This suggests that the early stimulation could make more significant contribution in enhancing the differentiation in 8 day cell growth.

The assay of CaN and NFAT (Fig. 9) showed that their changing tendency was almost same with one of the electrical stimulation dependent osteogenic differentiation activity (Fig. 4, 7 and 8), revealing that the electrical simulation derived up-regulation in osteogenic differentiation could be attributed to that the calcium ion signaling pathway was activated to trigger osteogenic differentiation.

Based on the mechanism of electrical stimulation on cellular function, the electric field of stimulation manipulates transmembrane potentials of cells and activates the voltage-gated calcium ion channels (VGICs). As a result, the fluxes of calcium ions through the activated VGICs induce the intracellular calcium ion oscillation, and the osteogenic differentiation is triggered.^35–38^ The VGICs have been proved to play an important role in enhancement of AD-MSCs’ and osteoblasts' functions of osteogenic differentiation during electrical stimulation.^36^ VGICs include high voltage-activated (HVA) channels that sub-classified into L-, N-, P-, Q- and R-types and low voltage-activated (LVA) channels (or T-type). VGICs to produce the biological function usually require specific stimulation voltage correspond to specific cells LVA channels are activated by lower membrane potentials, and HVA channels are opened in response to large membrane depolarizations and are activated at higher membrane potentials.^39,40^

In this study, the electrical simulation generated by *Q*_inj_ of 0.08 μQ showed the optimum effects in osteogenic differentiation, which might be attributed to that the studied *Q*_inj_ range from 0.03 μQ to 0.30 μQ for Ppy electrodes involves activation of different VGICs, and the stimulation with *Q*_inj_ of 0.08 μQ just activates a VGIC which can trigger well the osteogenic differentiation. The simulation to cells with high frequency of 50 Hz showed no role in promoting osteogenic differentiation (Fig. 7), it is speculated that the VGICs is activated too frequently to induce effective intracellular calcium ion oscillation, resulting in no activation of subsequent CaN/NFAT signaling pathway (Fig. 9). Since cell growth experiences adhesion, skeletal spreading, proliferation and differentiation, *etc.*, we suggest that the cells at different growth stage could have specific stimulation sensitivity to VGIC activation and intracellular calcium ion oscillation. The obvious enhancement in intracellular Ca^2+^ concentration at early stage may activate calcium ion pathway, whereas no effect on Ca^2+^ concentration with stimulation at later stage (Fig. 10). The cells at early growth stage could have good sensitivity and result in the early stimulation with more contributions to promoting cellular osteogenic differentiation.

## 5 Conclusion

Charge injection capacity (*C*_Q_) of polypyrrole (Ppy) electrodes could be tuned by electro-polymerization controlled Ppy thickness. The charge injection quantity (*Q*_inj_) onto the electrodes was a vital parameter and considered as stimulation pulse intensity to stimulate cells. The electrodes with *Q*_inj_ of 0.08 μQ resulted in the stimulation with a good effect in enhancing cellular osteogenic differentiation. The stimulation with 1 or 25 Hz showed an obvious role in improving the differentiation, and the early stimulation (day 2–5) in cell culture period of 8 days had more significant contribution to promote cellular osteogenic differentiation. These are attributed to activating of an appropriate VGIC and intracellular calcium ion oscillation, which induces up-regulation of the expression of osteogenesis-related gene and subsequently triggers osteogenic differentiation. Therefore, this study provides a way to regulate the osteogenic differentiation through electrical stimulation in the view of charge injection, a guide to design electrode in considering materials with *C*_Q_ and an alternative insight into understanding of the mechanism of electrical stimulation to cellular responses.

## Conflicts of interest

There are no conflicts to declare.

## Supplementary Material

RA-008-C8RA02601G-s001
