# Supplementary material for: Charge injection based electrical stimulation on polypyrrole planar electrodes to regulate cellular osteogenic differentiation
Source: RSC Adv. 2018 May 21;8(33):18470–9. doi: 10.1039/c8ra02601g (PMC9080618; doi:10.1039/c8ra02601g)
Supplement: RA-008-C8RA02601G-s001 [file RA-008-C8RA02601G-s001.pdf]

Electronic Supplementary Material (ESI) for RSC Advances.
This journal is © The Royal Society of Chemistry 2018

Supplementary Information

Charge injection based electrical stimulation on polypyrrole planar electrodes to regulate cellular osteogenic differentiation

Zongguang Liu[a], Lingqing Dong[a,b], Kui Cheng[a], Zhongkuan Luo[c], Wenjian Weng[a],*

a School of Materials Science and Engineering, State Key Laboratory of Silicon Materials, Zhejiang University, Hangzhou 310027, China

b The Affiliated Stomatologic Hospital, School of Medicine, Zhejiang University, Hangzhou 310003, China.

c Zhejiang-California International NanoSystems Institute, Hangzhou 310058, China

*Corresponding author: wengwj@zju.edu.cn

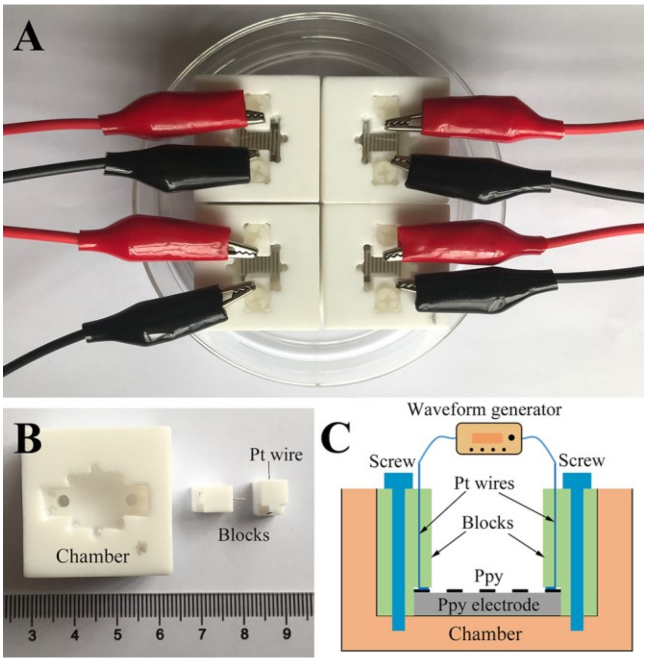

Fig. S1 The photos (A and B) and schematic (C) of the home-made culturing device used in this study. The device was consisted of four parts: (1) Culture chamber; (2) Pt wires; (3) Screws; and (4) Blocks used to press the Pt wires onto electrodes.

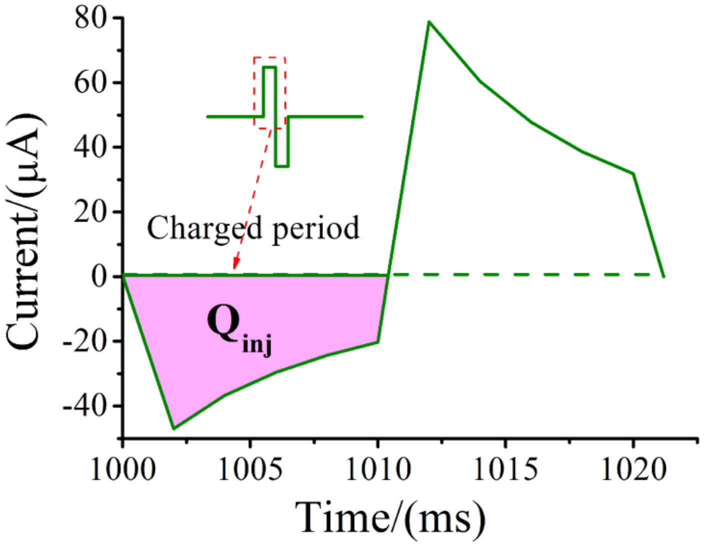

Fig. S2 The current-time curve was collected under a biphasic pulse signal with an applied voltage, illustrating the concept of charge injection quantity (Q_{inj}) (the total amount of charge injected on the electrode during a stimulus pulse). The current was integrated over time in the charged period in each curve using Originpro 8.5 software. The obtained integral area was the Q_{inj} on electrode.
